# Supplementary material for: Comparison of neural mobilization and conservative treatment on pain, range of motion, and disability in cervical radiculopathy: A randomized controlled trial
Source: PLoS One. 2022 Dec 6;17(12):e0278177. doi: 10.1371/journal.pone.0278177 (PMC9725158; doi:10.1371/journal.pone.0278177)

**INTRODUCTION**

Cervical Radiculopathy (CR) is a disorder of the spinal nerve roots that is largely caused by space occupying lesion, disc herniation compression and bony spur typically osteophytes in degenerating cervical spine which can lead to nerve root inflammation, impingement even both [^1^](#_ENREF_1). These lesions can trigger pain receptors in the soft tissues and joints of cervical spine that can lead to both sensory or the motor changes in upper extremity [^2^](#_ENREF_2) along with loss of sensation, numbness, tingling, fragility in upper end point, motor defects in neck and few times in scapula, and pain along the nerves pathway into the hand and arm, relying on where the affected roots are located.

The radiation of pain can be unilateral or bilateral, although bilateral cases occurred only in 5-36% of patients with cervical radiculopathy [^3^](#_ENREF_3)^,^ [^1^](#_ENREF_1). The upper quarter pain involves pain interpreted in the arm, shoulder, upper back, neck and upper chest area which might or not be associating with headache [^4^](#_ENREF_4).

The incidence of cervical radiculopathy annually is about 83.2 per 100 000 people. This incidence increase in the fifth decade of life that reaches to 203 per 100 000 [^3^](#_ENREF_3). It also results from roadside accidents, stenosis, overuse injuries of neck and exercise in bad posture. In 22% of patient’s disc involving diseases are main cause of CR, while combination of both diseases disc related and spondylitic changes include 68% patients. Nerve roots levels which are commonly affected in cervical radiculopathy are C6 and C7 [^5^](#_ENREF_5). Subjects with cervical radiculopathy although complain of neck pain but the main issue for which patient seeks therapy is pain in the arm [^3^](#_ENREF_3).

Among Pakistani population irregular physical activity is common, intensifying stress levels and deficiency of exercise are causing tremendous problems in daily routine. These factors result in bone, immune system and muscles feebleness as well as deteriorate body mechanics. Due to these factors, instability of the spine and related structures aggravate the underlying musculoskeletal problems [^6^](#_ENREF_6).

For establishing criteria for the diagnosis of cervical radiculopathy different diagnostic tests are devised. MRI and EMG are considered the gold standard for diagnosing cervical radiculopathy [^3^](#_ENREF_3). But these techniques are not readily available in most of clinical settings so other manual diagnostic tests are used. For that Wainner et al developed a criterion for diagnosis that is based on four variables. These include 1) Spurling test 2) upper limb tension test ULTT for median nerve 3) distraction test 4) cervical rotation. This clinical prediction rule has specificity of 94% and is useful method for the diagnosis of cervical radiculopathy [^1^](#_ENREF_1).

Cervical radiculopathy can be treated surgically but there is large number of evidence suggesting conservative management to be more effective than surgical treatment, suggesting multimodal treatment strategies that includes cervical traction, manual therapy techniques and strengthening exercises [^7^](#_ENREF_7)^,^ [^2^](#_ENREF_2). Although there is little high-quality evidence on the best non-operative therapy for cervical radiculopathy but these are used to relieve discomfort and pain [^8^](#_ENREF_8)^,^ [^9^](#_ENREF_9).

For this pathology conservative treatment options include therapeutic exercises for gaining strength and improving range of motion, manual therapy techniques along with mobilization and manipulation, use of different modalities such has heating, cryotherapy and traction, soft tissue mobilization techniques, medication and use of cervical collars [^10^](#_ENREF_10). In the present study strengthening exercises will be used as this regimen is successfully used as component of treatment program for patients with cervical radiculopathy [^11^](#_ENREF_11)^,^ [^12^](#_ENREF_12).

Another treatment used for the patients of cervical radiculopathy is neural mobilization technique [^7^](#_ENREF_7). Nervous system can undergo mechanical loads, for that it must be able to adapt and endure mechanical events of sliding, elongation, cross sectional changes, compression and angulation. But when the adaptation mechanism of nervous system fails it can undergo neural edema, fibrosis, hypoxia that can cause alteration in the neurodynamics of nervous system. This concept of neurodynamics by Shacklock is now more accepted term in physiotherapy than neural tension test, that examine the physical ability of the nervous system. This term refers to integrated biomechanical, physiological issues and also includes morphological functioning of the nervous system [^13^](#_ENREF_13)^,^ [^14^](#_ENREF_14). The extent of the neural movement depends on the surrounding tissues. These surrounding structures may impinge the passing nerve due to tightness or nerve may be sliding through the scars of these tissues or the nervous system may be overstretched, swollen and may have abnormal impulse generation[^14^](#_ENREF_14).

Neural mobilization is physical method for treatment of pain. This approach influences physiology of pain through mechanical treatment of neural tissues and the surrounding structures of nervous system. The main aim is to restore the disturbed dynamic balance between the neural tissues and surrounding non neural tissues. This will permit decrease pressure on the neural tissues and enhance optimal physiological functioning [^15^](#_ENREF_15)^,^ [^16^](#_ENREF_16). The benefits achieved through this include nerve gliding, reduced nerve adherence, improved vascularity of neural tissues, noxious fluids are dispersed and axoplasmic flow is improved[^14^](#_ENREF_14)^,^ [^16^](#_ENREF_16).

Neural mobilizations have been studied in various populations such as low back pain, carpal tunnel syndrome, lateral epicondalalgia and cervico-brachial pain [^13^](#_ENREF_13). Neural mobilization techniques studied include cervical lateral glides for cervico-brachial pain in which lateral translatory movement of c-spinal segment to be treated is done with an aim to move the structures surrounding the nerve [^17^](#_ENREF_17), nerve gliding exercises which are sequence of positioning of fingers to elongate median nerve used in the treatment of carpal tunnel syndrome [^18^](#_ENREF_18). However further researches are required for validation of this neural mobilization concept [^13^](#_ENREF_13). In the present study this approach will be used to evaluate its effects on patients with cervical radiculopathy.

Many studies have been done on treatment of cervical radiculopathy. But most of them are inconclusive in terms of defining appropriate treatment options that would be efficacious for the treatment of this pathology. As they lack in quality of study, number of subjects, treatment methods used, duration of sessions, measurements of outcomes, including patients with neck pain of multiple or unspecified origin or conditions masquerading as cervical radiculopathy and comparison of different treatments [^3^](#_ENREF_3)^,^ [^12^](#_ENREF_12) .

The purpose of this study is to assess the neural mobilization technique as an effective treatment for cervical radiculopathy through appropriate randomized controlled trial. Taking the factors and outcome measures under consideration that are not addressed properly in previous literature. As it is a cost effective technique, this study will help many people with cervical radiculopathy. Because of non-availability of different expensive modalities, these manual techniques will enhance the scope of rehabilitation in these subjects.

**OBJECTIVES**

The objective of the study was to compare the effectiveness of neural mobilization technique with conservative treatment on pain intensity, cervical range of motion, disability, cervical muscle endurance and quality of life

**methodology**

**Study design:**

Randomized controlled trial.

**Study setting:**

Physiotherapy department of Mayo Hospital, Lahore.

**Duration of study:**

18 months.

**Sample size:**

88 patients (44 in Group A and 44 in group B) will be included in the study using effect size 0.70, calculated from the parent article using (mean±SD) in group 1 (3.35±1.49) and group 2 (4.45±1.63) ^26^, at level of significance 0.05 and power 90% calculated by using G* Power [^19^](#_ENREF_19).

**Formula**:


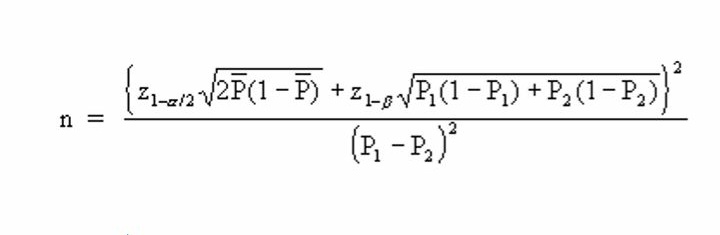


**Sampling technique:**

Convenience sampling.

**Sampling criteria:**

**Inclusion criteria:**

- Age group between 35-50 years
- Gender both male and female
- Subjects having radiating symptoms of cervical radiculopathy
- Subjects with no previous cervical surgeries
- Subjects with no loss of the upper limb movement

**Exclusion criteria:**

- Subjects having traumatic history
- Subjects with Osteoporosis
- Hypermobile patients
- Subjects with circulatory disturbances
- Subjects with peripheral nerve entrapment
- Subjects with tumor causing cervical radiculopathy
- Patients who are not willing to be included in the study.

# **ETHICAL CONSIDERATIONS**

1. It will be gained prior from the Ethical Committee of the University before study
2. Written informed consent will be taken from all the patients
3. All information and collected data will be kept confidential.
4. Participants will remain anonymous throughout the study.
5. The subjects will be informed that there are no disadvantages or risks on the procedure of the study.
6. They will also be informed that they will be free to withdraw at any time during the process of the study.

**DATA COLLECTION PROCEDURE:**

This randomized trial will be conducted according to consolidated standards of reporting trial CONSORT guidelines (2010) [^20^](#_ENREF_20). This will be parallel group randomized controlled trial with 1:1 allocation ratio into two groups. Study will be conducted at physical therapy department of Mayo Hospital Lahore.

In order to recruit the patients in the study. Patients between ages 35-50 years, willing to participate will be chosen according to inclusion criteria. Patients will be asked to sign a consent form and give their will regarding being enrolled in the study.

**Randomization:**

Randomization sequence will be created by using Excel 2016 with a 1:1 allocation using simple randomization by an independent researcher. Patients will be divided into two treatment groups, group A and group B randomly.

Patients will be allocated to two groups by concealment of allocation through sealed envelopes.

## Concealment of allocation:

Allocation concealment will be achieved with sequentially numbered, opaque, sealed, envelopes SNOSE. SNOSE will be used according to guidelines of Doig and Simpson [^21^](#_ENREF_21).

An independent researcher with no clinical involvement in the trial will make the concealed envelopes. 88 Envelopes will be made. Half envelops will contain folded papers with Treatment A written on them and the remaining half will contain folded papers with Treatment B written on them. A carbon paper will be inserted in each envelop with carbon side facing the paper so the allocation sequence, patient name, date of birth of participant and other information can be transferred onto allocation paper inside the envelope.

A piece of tin foil is also inserted into envelop so the treatment card cannot be read against light. Envelops will be sealed and signed by the maker. A unique randomized number will be allocated to these envelops and shuffled vigorously. Then the envelops will be arranged sequentially and handed over to another independent researcher.

Assessor will pretest the participant and if eligible envelope will be allocated to subject. Therapist will record the information on the envelope and open it afterwards to maintain the concealment. Assessor will record the post treatment findings and another independent analyst will analyze the data.

This allocation of concealment will ensure the unpredictability of treatment allocation by investigators and patients.

## Blinding:

In this study patients, assessors, data analysts will be blinded to allocation of treatment groups in this study. Except for the therapist all other staff will be kept blinded as they will not be informed about the details of allocation. Trial will be adhered to established procedures to maintain separation between staff who will collect outcome measurements and the therapist. Patient will be blinded to treatment allocation as treatment will be given in separate rooms for each group. Therapist who is not blinded will not take the outcome measurements. All the other assessors, investigators and analysts will not know the details of treatment.

**Group A (Experimental group):**

In group A neural mobilization technique with sliding of median nerve will be applied with 3 seconds hold in each repetition, Neural mobilization will be done according to technique described by David Butler [^22^](#_ENREF_22). Subject will be placed in supine position and slider neural mobilization of the median nerve will be given. In this group conservative treatment which will include cervical isometrics exercises with 10 repetitions in each direction with 5 seconds hold will also be given. Isometric exercises will be performed with the patient in sitting position.

**Group B (control group):**

In group B conservative treatment will be given which will include cervical isometrics exercises with 10 repetitions in each direction with 5 seconds hold will also be given. Isometric exercises will be performed with the patient in sitting position 3 sets of these exercises will be performed with the rest period of 30seconds.

All the subjects will be given hot packs for 10 minutes prior to the treatment.

Subjects having the same baseline will be given treatment for 12 sessions (3 times per week for 4 weeks). Pre assessment will be done at baseline, second assessment will be done after 2 weeks and final post assessment will be done at the end of 12^th^ session in 4^th^ week.

**Diagnosis:**

For the diagnosis of the patients with cervical radiculopathy clinical predictor rule will be applied consisting of Spurling’s test, Distraction test, ULNTT for median nerve and ipsilateral neck rotation [^23^](#_ENREF_23). Radiological imaging such as MRI will also be used for diagnosis.

**Constraints:**

Patient regularity and drop out might be a major constraint as my patient population ranges between age of 35-50, individuals in this age have a busy work routine hence once patient feels a little relief, he feels boredom visiting hospital or often leave from work place is an issue, however this issue will be tried to resolve by asking them about their will to complete the study and writing an official letter to their work place if needed and applicable.

# **Outcome measures:**

# The main outcome will be the effectiveness of neural mobilization technique on pain intensity measured on VAS, endurance measured by cranio cervical flexion test and range of motion measured on inclinometer. Secondary outcomes will be to see the effectiveness of conservative treatment and measuring the effects of treatment on quality of life [^24^](#_ENREF_24).

## Baseline measurements:

Patient age, gender, occupation, duration of pain, hypertension, heart disease, smoking and diabetes mellitus history will be noted at the time of recruitment. Further baseline pain, ROM, endurance, quality of life will also be noted.

## Visual analogue scale:

Visual analogue scale will be used to assess level of pain before, after and during the course of study.

## Inclinometer:

Inclinometer will be used to measure the ROM.

## Neck disability index (NDI):

Neck disability will be assessed by using neck disability index [^24^](#_ENREF_24).

## Cranio-cervical flexion test:

Cranio-cervical flexion test will be used to assess the neck endurance

Measurements will be taken at three levels before treatment, during treatment and at the end of treatment and a follow up reading to confirm the long lasting effect of the respective treatment [^25^](#_ENREF_25).

**SF-36:**

Improvement in quality of life will be assessed by using SF-36. That includes physical functioning, bodily pain, role limitations due to physical health problems, role limitations due to personal or emotional problems, emotional well-being, social functioning, energy/fatigue, and general health perceptions [^26^](#_ENREF_26)^,^ [^27^](#_ENREF_27).

**Data analysis procedure:**

Data will be analyzed on SPSS version 21 and MS Excel 2010. Descriptive analysis (mean, median, mode, variance, standard deviation) will be performed for quantitative data i.e. continuous and discrete variables. Frequencies and percentages will be calculated for categorical and nominal data. For graphical representation, continuous and discrete variables will be presented with histograms with normal curve and categorical and ordinal data will be presented in form of bar and pie charts.

Independent samples t-test and Man Whitney test will be used accordingly for between group comparisons. Repeated measure ANOVA will be used for within group analysis.

Inferential statistics will include independent samples t test and Man Whitney test at various levels of assessment for between group comparison while Freidman and repeated measure ANOVA will be performed for within group analysis separately. Excel 2010 will be used for scatter plotting and routine calculations.

**CONSENT FORM (ENGLISH)**

“EFFECTIVENESS OF NEURO MOBILIZATION TECHNIQUE ON PAIN, RANGE OF MOTION, MUSCLE ENDURANCE AND DISABILITY IN CERVICAL RADICULOPATHY, A RANDOMIZED CONTROLLED TRIAL”

**Description of the research and your participation:**

You are invited to participate in a research study conducted by SHAZIA RAFIQ. The purpose of this research is to find the treatment protocol among subjects with cervical radiculopathy. Your participation will be involved to complete the study.

**Risks and discomforts:**

There are no known risks associated to these treatment options.

**Potential benefits:**

There are known benefits of this research to find out if this treatment protocol helps to improve quality of life, range of motion and pain in cervical radiculopathy. So then the same measures can be used for other patients.

**Protection of confidentiality:**

We will do everything we can to protect your privacy. Your identity will not be revealed in any publication resulting from this study.

**Voluntary participation:**

Your participation in this research study is voluntary. You may choose not to participate and you may withdraw your consent to participate at any time. You will not be penalized in any way should you decide not to participate or to withdraw from this study.

**Contact information:**

If you have any questions or concerns about this study or if any problems arise, please contact SHAZIA RAFIQ, Ph.D. scholar of University Institute of Physical Therapy at University of Lahore, Mobile no.0321-4012898 and University of Lahore at 0092-42-111-865865 ext-2802. If you have any questions or concerns about your rights as a research participant, please contact the University Institutional Review Board.

**Consent:**

I have read this consent form and have been given the opportunity to participate in the research work. I give my consent to participate in this study.

Participant’s signature_______________________________ Date:_______________

# **RESEARCH WORK PLAN**

Analysed (n = …)

Excluded from analysis (give reasons) (n = …)

Lost to follow-up (give reason) (n = …)

Discontinued intervention (give reasons) (n = …)

# Allocation:

# Care Providers

# Allocation :

# Patients

Assessed for eligibility=88

(n = …)

Allocated to intervention (n =44)

Received allocated intervention (n =44)

Did not receive allocated intervention (give reasons) (n = …)

Care providers (n = 1 ), teams (N = 5 ), centers (n= 1 ) performing the intervention

Care providers (n = 1 ), teams (n = 5), centers (n= 1 ) performing the intervention

Allocated to intervention (n =44)

Received allocated intervention (n =44)

Did not receive allocated intervention (give reasons) (n = …)

**Analysis**

**Patients**

**Follow-up**

**Patients**

# Enrollment

# Patients

Analysed (n = …)

Excluded from analysis (give reasons) (n = …)

Lost to follow-up (give reason) (n = …)

Discontinued intervention (give reasons) (n = …)

Excluded (n = …)

- Not meeting inclusion criteria (n = …)
- Refused to participate (n = …)
- Other reason (n = …)

Randomized (n =88)

1. **References**

1. Wainner RS, Fritz JM, Irrgang JJ, Boninger ML, Delitto A, Allison S. Reliability and diagnostic accuracy of the clinical examination and patient self-report measures for cervical radiculopathy. *Spine.* 2003;28(1):52-62.

2. Boyles R, Toy P, Mellon J, Hayes M, Hammer B. Effectiveness of manual physical therapy in the treatment of cervical radiculopathy: a systematic review. *Journal of Manual & Manipulative Therapy.* 2011;19(3):135-142.

3. Cleland JA, Whitman JM, Fritz JM, Palmer JA. Manual physical therapy, cervical traction, and strengthening exercises in patients with cervical radiculopathy: a case series. *Journal of Orthopaedic & Sports Physical Therapy.* 2005;35(12):802-811.

4. Elvey R, Hall T. Neural tissue evaluation and treatment. *Physical therapy of the shoulder*; 2011: 187-203.

5. Sambyal S, Kumar S. Comparison Between Nerve Mobilization And Conventional Physiotherapy In Patients With Cervical Radiculopathy. *International Journal of Innovative Research and Development.* 2013;2(8).

6. Umar M, Naeem A, Badshah M, Amjad I. Effectiveness of cervical traction combined with core muscle strengthening exercises in cervical radiculopathy: a randomized control trial. *J Public Health Biol Sci.* 2012;1:115-120.

7. Murphy DR, Hurwitz EL, Gregory A, Clary R. A nonsurgical approach to the management of patients with cervical radiculopathy: a prospective observational cohort study. *Journal of manipulative and physiological therapeutics.* 2006;29(4):279-287.

8. Cheng C-H, Tsai L-C, Chung H-C, et al. Exercise training for non-operative and post-operative patient with cervical radiculopathy: a literature review. *Journal of physical therapy science.* 2015;27(9):3011-3018.

9. Bertozzi L, Gardenghi I, Turoni F, et al. Effect of therapeutic exercise on pain and disability in the management of chronic nonspecific neck pain: systematic review and meta-analysis of randomized trials. *Physical therapy.* 2013;93(8):1026-1036.

10. Wainner RS, Gill H. Diagnosis and nonoperative management of cervical radiculopathy. *Journal of Orthopaedic & Sports Physical Therapy.* 2000;30(12):728-744.

11. Waldrop MA. Diagnosis and treatment of cervical radiculopathy using a clinical prediction rule and a multimodal intervention approach: a case series. *Journal of Orthopaedic & Sports Physical Therapy.* 2006;36(3):152-159.

12. Costello M. Treatment of a patient with cervical radiculopathy using thoracic spine thrust manipulation, soft tissue mobilization, and exercise. *Journal of Manual & Manipulative Therapy.* 2008;16(3):129-135.

13. Ellis RF, Hing WA. Neural mobilization: a systematic review of randomized controlled trials with an analysis of therapeutic efficacy. *Journal of Manual & Manipulative Therapy.* 2008;16(1):8-22.

14. Butler DS. *The sensitive nervous system*: Noigroup publications; 2000.

15. Shacklock M. Neurodynamics. *Physiotherapy.* 1995;81(1):9-16.

16. Shacklock M. *Clinical neurodynamics: a new system of musculoskeletal treatment*: Elsevier Health Sciences; 2005.

17. Coppieters MW, Stappaerts KH, Wouters LL, Janssens K. The immediate effects of a cervical lateral glide treatment technique in patients with neurogenic cervicobrachial pain. *Journal of Orthopaedic & Sports Physical Therapy.* 2003;33(7):369-378.

18. Coppieters MW, Alshami AM. Longitudinal excursion and strain in the median nerve during novel nerve gliding exercises for carpal tunnel syndrome. *Journal of orthopaedic Research.* 2007;25(7):972-980.

19. Faul F, Erdfelder E, Lang A-G, Buchner A. G* Power 3: A flexible statistical power analysis program for the social, behavioral, and biomedical sciences. *Behavior research methods.* 2007;39(2):175-191.

20. Schulz KF, Altman DG, Moher D. CONSORT 2010 statement: updated guidelines for reporting parallel group randomised trials. *BMC medicine.* 2010;8(1):18.

21. Doig GS, Simpson F. Randomization and allocation concealment: a practical guide for researchers. *Journal of critical care.* 2005;20(2):187-191.

22. Butler DS. *The neurodynamic techniques: a definitive guide from the Noigroup team*: Noigroup publications; 2005.

23. Savva C, Giakas G. The effect of cervical traction combined with neural mobilization on pain and disability in cervical radiculopathy. A case report. *Manual therapy.* 2013;18(5):443-446.

24. Cleland JA, Childs JD, Whitman JM. Psychometric properties of the Neck Disability Index and Numeric Pain Rating Scale in patients with mechanical neck pain. *Archives of physical medicine and rehabilitation.* 2008;89(1):69-74.

25. Jull GA, O'leary SP, Falla DL. Clinical assessment of the deep cervical flexor muscles: the craniocervical flexion test. *Journal of Manipulative & Physiological Therapeutics.* 2008;31(7):525-533.

26. Côté P, Cassidy JD, Carroll LJ, Kristman V. The annual incidence and course of neck pain in the general population: a population-based cohort study. *Pain.* 2004;112(3):267-273.

27. de Vries GE, Jorritsma W, Dijkstra PU, Geertzen JH, Reneman MF. The construct validity of the Short Form-36 Health Survey for patients with nonspecific chronic neck pain. *International Journal of Rehabilitation Research.* 2015;38(2):137-143.

**questionnaire**

**SECTION 1**

**Name___________________ Study Serial No: _____________**

**Gender:** Male/ Female **Date: _______________**

**Age (Years): ______________ Occupation: ___________________________**

**Previous History of Neck Injury:** ⃝ Yes ⃝ No

**Hypertension:** Yes/No **Diabetes :** Yes/No

**Heart Disease:** Yes/No **Smoking:** Yes/ No **Surgical History: _________________**

**SECTION 2**

**NECK DISABILITY INDEX:**

This questionnaire is designed to enable us to understand how much your neck pain has affected your ability to manage in everyday life. Please answer every section and mark in each section only once, which applies to you. We realize you may consider that two of the statements in any section may relate to you, but please just mark one which most closely described your problem.

| **Section 1 : Pain Intensity** | **Pre** | **Post** |
| --- | --- | --- |
| 0. I have no pain at the movement. |  |  |
| 1. The pain is very mild at the movement. |  |  |
| 2. The pain is moderate at the movement. |  |  |
| 3. The pain is fairly severe at the movement. |  |  |
| 4. The pain is very severe at the movement. |  |  |
| 5. The pain is the worst imaginable at the movement. |  |  |
| **Section 2 : Personal Care** |  |  |
| 0. I can look after myself normally without causing extra pain. |  |  |
| 1. I can look after myself normally but it causes extra pain. |  |  |
| 2. It is painful to look after myself and I am slow and careful. |  |  |
| 3. I need some help but manage most of my personal care. |  |  |
| 4. I need help everyday in most aspects of self care. |  |  |
| 5. I do not get dressed; I wash with difficulty and stay in bed. |  |  |
| **Section 3 : Lifting (Skip if you have not attempted lifting since the onset of your neck pain)** |  |  |
| 0. I can lift heavy weights without extra pain. |  |  |
| 1. I can lift heavy weights but it gives extra pain. |  |  |
| 2. Pain prevents me from lifting heavy weights off the floor, but I can manage if they are conveniently positioned eg. On a table. |  |  |
| 3. Pain prevents me from lifting heavy weights but I can manage light to medium weight if they are conveniently positioned. |  |  |
| 4. I can only lift very light weights. |  |  |
| 5. I cannot lift or carry anything at all. |  |  |

| **Section 4 : Reading** |  |  |
| --- | --- | --- |
| 0. I can read as much as I want to with no pain in my neck. |  |  |
| 1. I can read as much as I want to with slight pain in my neck. |  |  |
| 2. I can read as much as I want with moderate pain in my neck. |  |  |
| 3. I cannot read as much as I want because of moderate pain in my neck. |  |  |
| 4. I can hardly read at all because of severe pain in my neck. |  |  |
| 5. I cannot read at all. |  |  |
| **Section 5 : Headache** |  |  |
| 0. I have no headaches at all. |  |  |
| 1. I have slight headaches that come infrequently. |  |  |
| 2. I have moderate headaches which come infrequently. |  |  |
| 3. I have moderate headaches which come frequently. |  |  |
| 4. I have severe headaches which come frequently |  |  |
| 5. I have headache almost all the time. |  |  |
| **Section 6 : Concentration** |  |  |
| 0. I can concentrate fully when I want to with no difficulty. |  |  |
| 1. I can concentrate fully when I want to with slight difficulty. |  |  |
| 2. I have a fair degree of difficulty in concentrating when I want to. |  |  |
| 3. I have a lot of difficulty in concentrating when I want to. |  |  |
| 4. I have a great deal of difficulty in concentrating when I want to. |  |  |
| 5. I cannot concentrate at all. |  |  |
| **Section 7 : Work** |  |  |
| 0. I can do as much work as I want to. |  |  |
| 1. I can do my usual work, but no more. |  |  |
| 2. I can do most of my usual work, but no more. |  |  |
| 3. I cannot do my usual work. |  |  |
| 4. I can hardly do any work at all. |  |  |
| 5. I cannot do any work at all. |  |  |

|  | | |
| --- | --- | --- |
| **Section 8 : Driving** |  |  |
| 0. I can drive my car without any neck pain. |  |  |
| 1. I can drive my car as long as I want with slight pain in my neck. |  |  |
| 2. I can drive my car as long as I want with moderate pain in my neck. |  |  |
| 3. I cannot drive my car as long as I want because of moder |  |  |
| 4. I can hardly drive at all because of severe pain in my neck. |  |  |
| 5. I cannot drive my car at all. |  |  |
| **Section 9 : Sleeping** |  |  |
| 0. I have no trouble sleeping. |  |  |
| 1. My sleep is slightly disturbed (less than 1 hr sleepless). |  |  |
| 2. My sleep is mildly disturbed (1-2 hrs of sleepless). |  |  |
| 3. My sleep is moderately disturbed (2-3 hrs of sleepless). |  |  |
| 4. My sleep is greatly disturbed (3-5 hrs of sleepless). |  |  |
| 5. My sleep is completely disturbed (5-7 hrs of sleepless). |  |  |
| **Section 10 : Recreation** |  |  |
| 0. I am able to engage in all my recreation activities with no neck pain at all. |  |  |
| 1. I am able to engage in all my recreation activities, with some pain in my neck. |  |  |
| 2. I am able to engage in most, but not all of my recreation activities because of pain in my neck. |  |  |
| 3. I am able to engage in a few of my usual recreation activities because of pain in my neck |  |  |
| 4. I can hardly do any recreation activities because of pain in my neck. |  |  |
| 5. I cannot do any recreation activities at all. |  |  |
| **Total score** |  |  |

**NDI scoring**

Each of the 10 sections is scored separately (0 to 5 points each) and then added up (max.total=50) .If all 10 sections are complete, simply double the patients score.

If a section is omitted, divide the patient’s total score by the number of sections completed times 5.

FORMULA: PATIENT’S SCORE X 100 = ___________% DISABILITY

# OF SECTIONS COMPLETED X 5

Pre treatment score :_____________ Post treatment score:____________

**NDI SCORING**

Each of the 10 sections is scored separately (0 to 5 points each) and then added up

(max.total=50)

If all 10 sections are complete, simply double the patients score.

If a section is omitted, divide the patient’s total score by the number of sections

completed times 5.

FORMULA: PATIENT’S SCORE X 100 = ___________% DISABILITY

# OF SECTIONS COMPLETED X 5

**Functional Assessment by Neck Disability Index:**

Pre :

Post:

**SECTION 3**

**NUMERIC RATING SCALE (NRS):**

Try and assign a number from 0 to 10 to your current pain level. If you have no pain, use a 0. As the numbers get higher, they stand for pain that is getting worse. A 10 means the pain is as bad as it can be.

| **PRE SCORE :** |
| --- |

| **POST SCORE :** |
| --- |


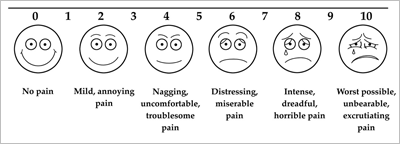


**SECTION 4**

**RANGE OF MOTION BY INCLINOMETER:**

| Outcome Measure | Measurement | Pre treatment  Assessment | Post treatment  Assessment |
| --- | --- | --- | --- |
| ROM of cervical spine | Flexion:  Extension:  Side bending (R):  Side bending  (L):  Rotation (R):  Rotation (L): |  |  |
|  |  |  |  |

**SECTOION 5**

**Craniocervical neck flexion:**

| **Reading** | **Time** | **Range of motion/level of inflation of stabalizer** |
| --- | --- | --- |
| **Pre treatment** | **10 seconds** |  |
| **Mid treatment** | **10 seconds** |  |
| **Post treatment** | **10 seconds** |  |

**SECTION 6**


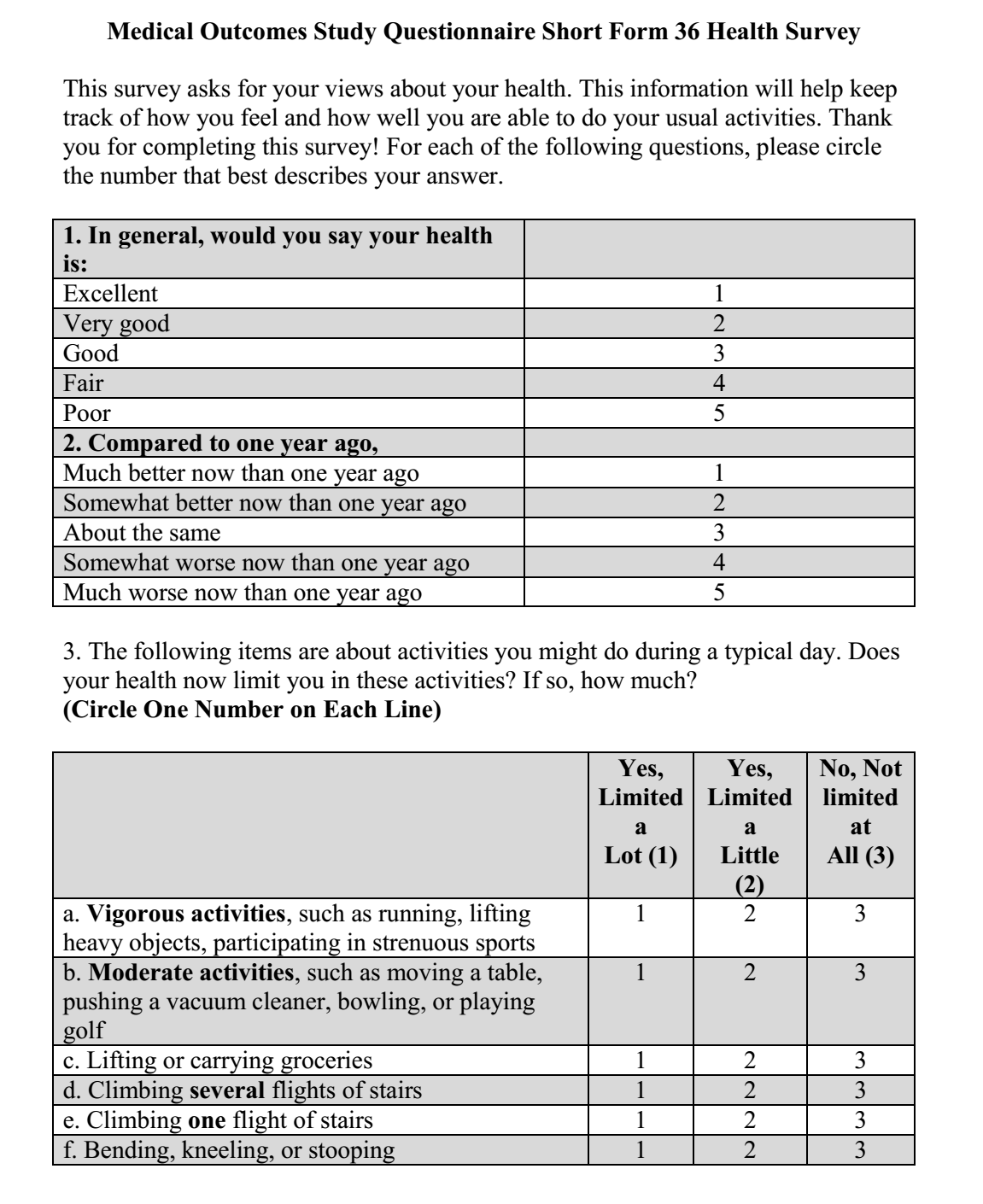


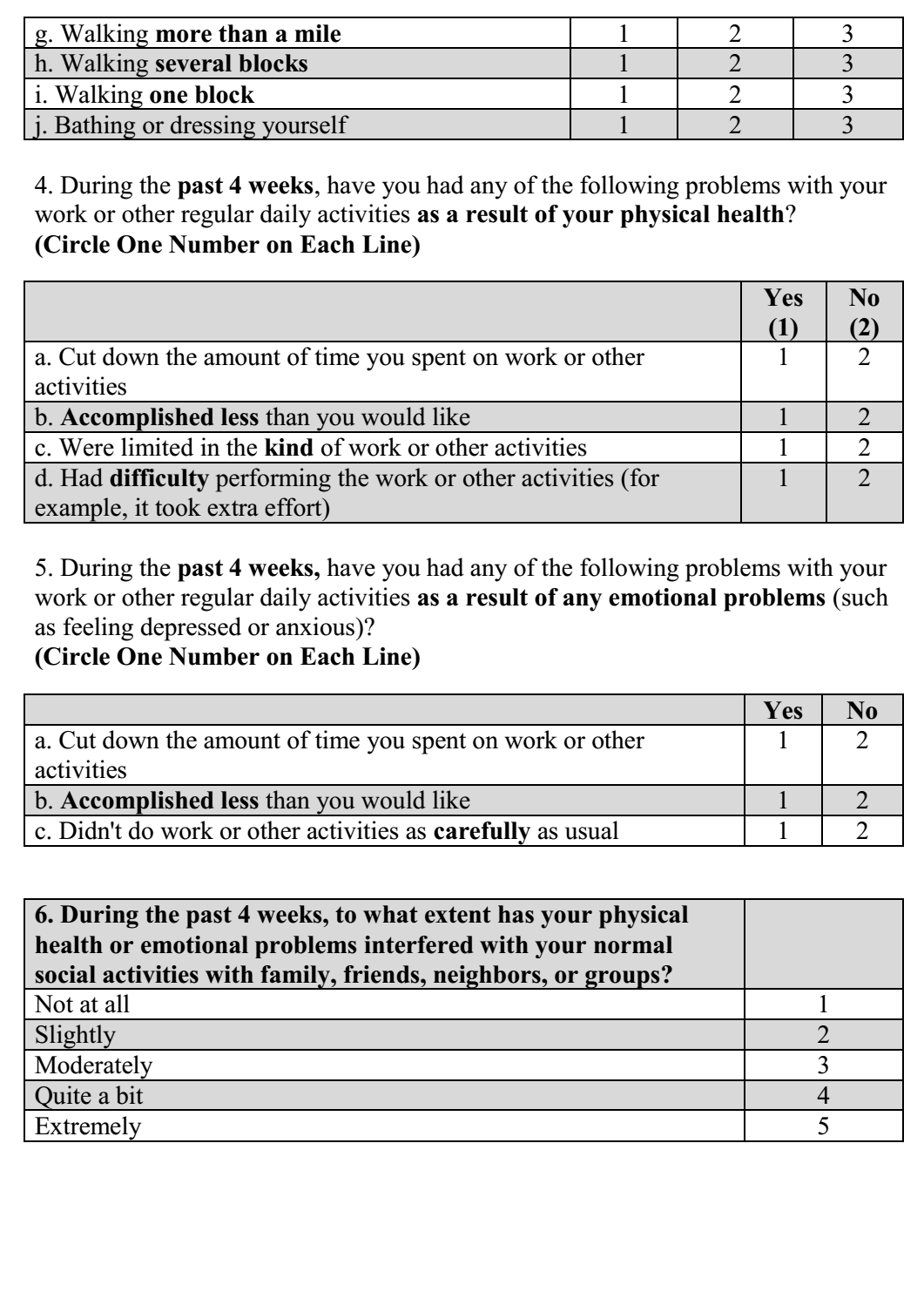


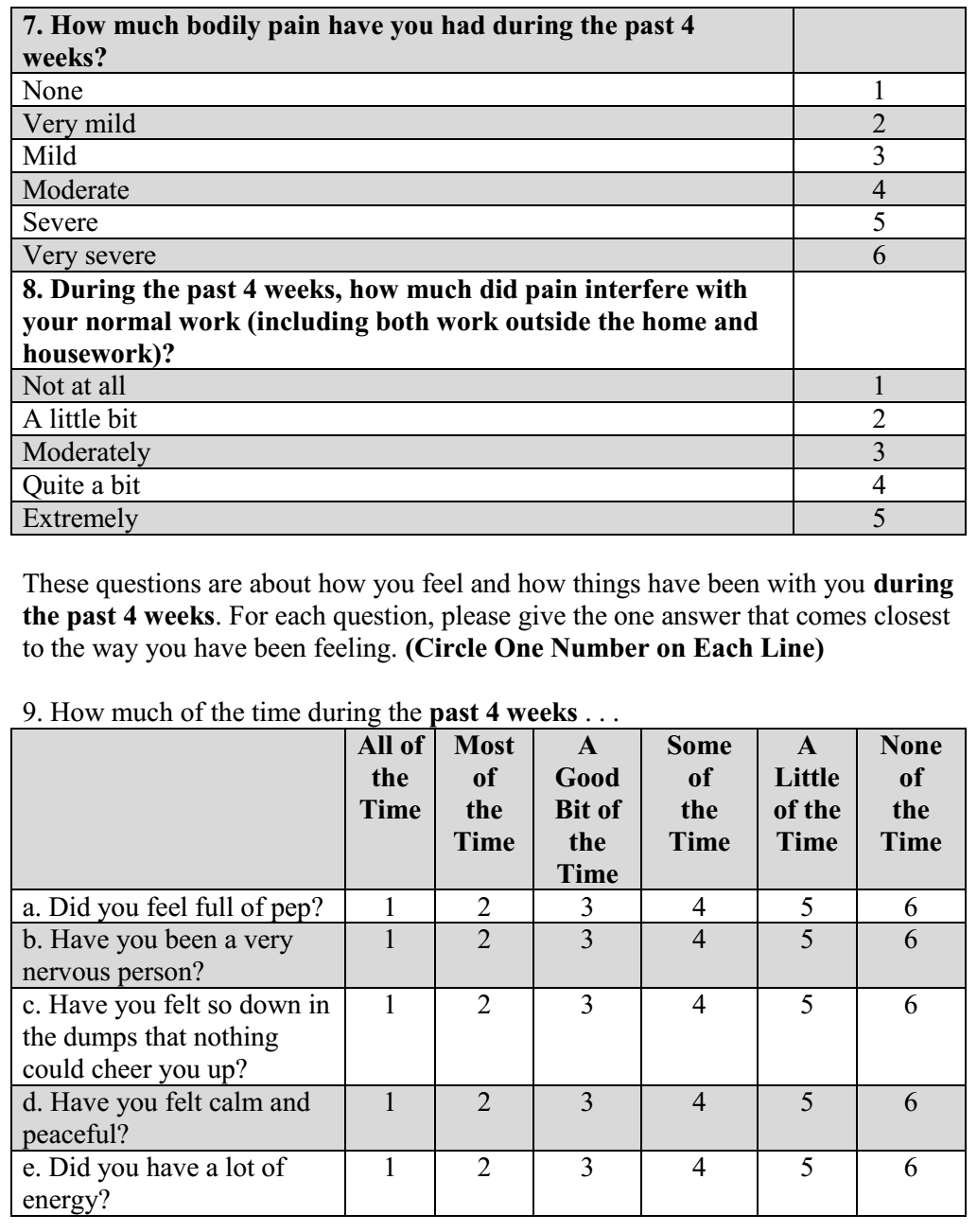


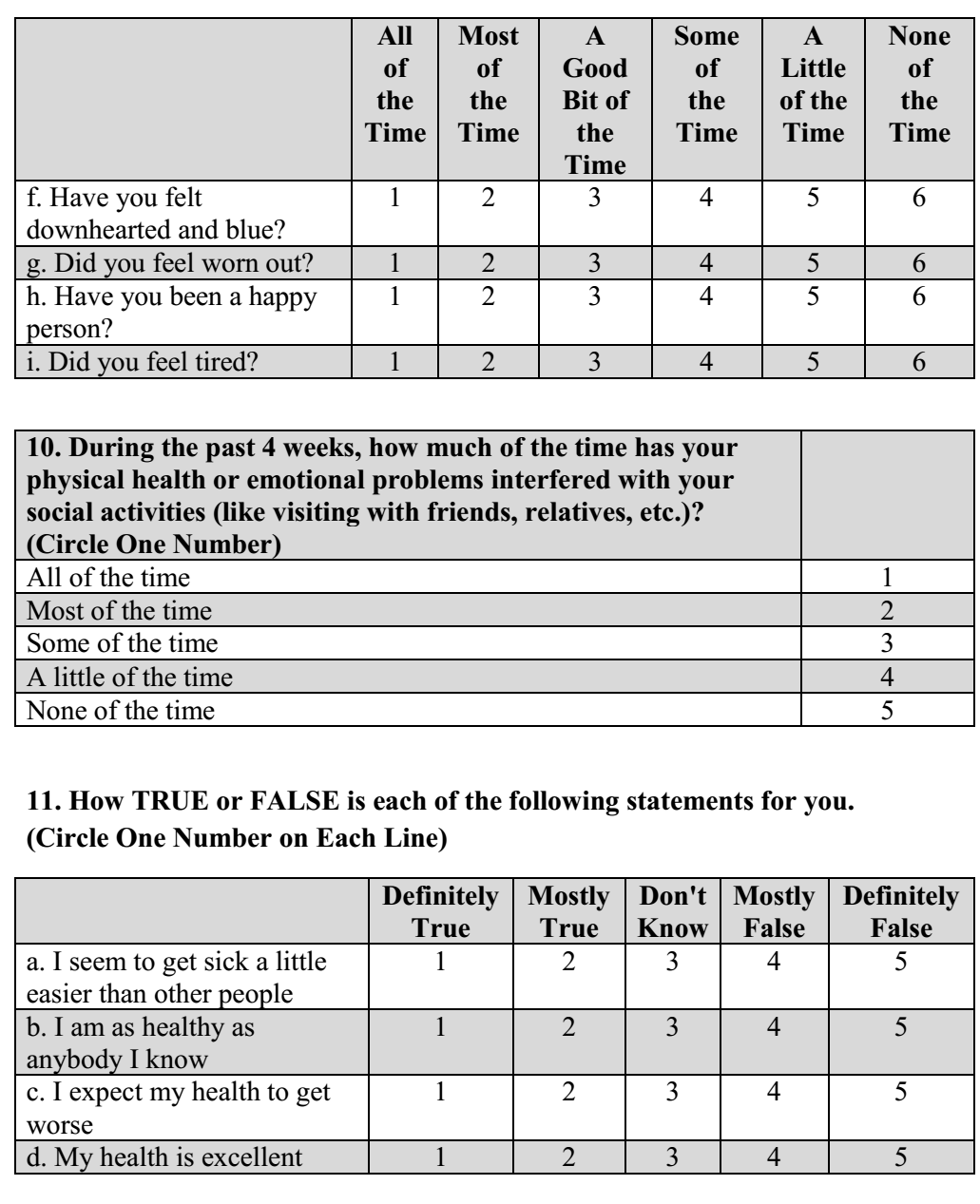

Supplement: S1 File — (DOCX) [file pone.0278177.s002.docx]
