# Supplementary material for: Comparison of neural mobilization and conservative treatment on pain, range of motion, and disability in cervical radiculopathy: A randomized controlled trial
Source: PLoS One. 2022 Dec 6;17(12):e0278177. doi: 10.1371/journal.pone.0278177 (PMC9725158; doi:10.1371/journal.pone.0278177)
Supplement: Attachment — Submitted filename: Reviwer COMMENTS.docx [file pone.0278177.s005.docx]

I am grateful to the reviewers for their insightful comments on the paper. We have been able to incorporate changes to reflect most of the suggestion provided by the reviewers. Here in the red color text is a point-by-point response to the reviewer’s comments and concerns.

When submitting your revision, we need you to address these additional requirements.

1. Please ensure that your manuscript meets PLOS ONE's style requirements, including those for file naming. The PLOS ONE style templates can be found at

Guidelines has been followed during development of manuscripts.

2. We noticed you have some minor occurrence of overlapping text with the following previous publication, which needs to be addressed:

Reference of the article has been given in methodology.

3. Thank you for stating the following financial disclosure:

No specific funding was received for this research.

4. We note that you have indicated that data from this study are available upon request. PLOS only allows data to be available upon request if there are legal or ethical restrictions on sharing data publicly. For information on unacceptable data access restrictions, please see http://journals.plos.org/plosone/s/data-availability#loc-unacceptable-data-access-restrictions.

All relevant data is available within the manuscript. Data cannot be shared publicly because of ethical concerns. Patients were included on a no objection base to conduct the study. Patients were not asked for permission to publish full encrypted data.

In your revised cover letter, please address the following prompts:

a) If there are ethical or legal restrictions on sharing a de-identified data set, please explain them in detail (e.g., data contain potentially identifying or sensitive patient information) and who has imposed them (e.g., an ethics committee). Please also provide contact information for a data access committee, ethics committee, or other institutional body to which data requests may be sent.

b) If there are no restrictions, please upload the minimal anonymized data set necessary to replicate your study findings as either Supporting Information files or to a stable, public repository and provide us with the relevant URLs, DOIs, or accession numbers. Please see http://www.bmj.com/content/340/bmj.c181.long for guidelines on how to de-identify and prepare clinical data for publication. For a list of acceptable repositories, please see http://journals.plos.org/plosone/s/data-availability#loc-recommended-repositories.

We will update your Data Availability statement on your behalf to reflect the information you provide.

Data availability statement has been updated on the cover page

5. We noted in your submission details that a portion of your manuscript may have been presented or published elsewhere. Please clarify whether this [conference proceeding or publication] was peer-reviewed and formally published. If this work was previously peer-reviewed and published, in the cover letter please provide the reason that this work does not constitute dual publication and should be included in the current manuscript.

This article is present in the pre-prints and was mentioned in the cover letter and link is as follow <https://www.researchsquare.com/article/rs-223383/v1> . One other article containing different variables were extracted from this research and is published recently in BioMed journal; link is as follow https://www.hindawi.com/journals/bmri/2022/9385459

6. Your ethics statement should only appear in the Methods section of your manuscript. If your ethics statement is written in any section besides the Methods, please delete it from any other section.

Remove it from abstract and check in methodology

Reviewers' comments:

Reviewer's Responses to Questions

**Comments to the Author**

1. Is the manuscript technically sound, and do the data support the conclusions?

The manuscript must describe a technically sound piece of scientific research with data that supports the conclusions. Experiments must have been conducted rigorously, with appropriate controls, replication, and sample sizes. The conclusions must be drawn appropriately based on the data presented.

Reviewer #1: Yes

Reviewer #2: Partly

2. Has the statistical analysis been performed appropriately and rigorously?

Reviewer #1: Yes

Reviewer #2: I Don't Know

3. Have the authors made all data underlying the findings in their manuscript fully available?

The [PLOS Data policy](http://www.plosone.org/static/policies.action#sharing) requires authors to make all data underlying the findings described in their manuscript fully available without restriction, with rare exception (please refer to the Data Availability Statement in the manuscript PDF file). The data should be provided as part of the manuscript or its supporting information, or deposited to a public repository. For example, in addition to summary statistics, the data points behind means, medians and variance measures should be available. If there are restrictions on publicly sharing data—e.g. participant privacy or use of data from a third party—those must be specified.

Reviewer #1: Yes

Reviewer #2: Yes

4. Is the manuscript presented in an intelligible fashion and written in standard English?

PLOS ONE does not copyedit accepted manuscripts, so the language in submitted articles must be clear, correct, and unambiguous. Any typographical or grammatical errors should be corrected at revision, so please note any specific errors here.

Reviewer #1: Yes

Reviewer #2: Yes

5. Review Comments to the Author

Please use the space provided to explain your answers to the questions above. You may also include additional comments for the author, including concerns about dual publication, research ethics, or publication ethics. (Please upload your review as an attachment if it exceeds 20,000 characters)

Reviewer #1: Please make the following modifications to the paper:
1. Please revise the study title in light of the current study's objectives.

Study title has been revised.

2. Work on balancing the different sections of the research abstract, as the method and results sections are clearly overly detailed.

Method and results sections are revised.

3. I hope the author(s) will be as brief as possible in these two sections of the abstract - methods and results - with a focus on the most important research findings in the results section of the study abstract.

Method and results sections in abstract are revised.

4. The introduction is far too long and detailed. I hope to limit the introduction to two or three paragraphs at most, with the final paragraph devoted to the current research problem and how to solve it within the framework of the current study's design objective.

Information in introduction section has been curtailed.

5. I noticed that the material and method sections were written as paragraphs with side headings. The names of the side headings, in my opinion, are ineffective in describing what is related to them in this section, which the author(s) should change.

Methodology of other published articles in PLOS one is carefully reviewed. In this article subheadings are given where necessary to describe details of methodology.

6. Please relate Figure 1 to the texts in the material and methods section.

Figure 1 has been mentioned in the text of material and methods.

7. The author(s) should carefully review the progress of their research results to avoid the presence of statistical flaws, some of which were discovered while reviewing the paper, with the goal of displaying graphic images to alleviate the boredom caused by displaying the results only in the style of statistical tables.

I have carefully reviewed the result section but were unable to find the statistical flaws. It would be of great if you could point out what kind of flaws has been noticed.

8. Author(s) should also avoid using side titles in the results section, which is not recommended.

Side titles in the results section has been removed.

9. I noticed that the discussion section served as an introduction to the study, and the discussion appeared to be completely inadequate in terms of proving or denying the current study's findings with previous studies, so please review this shortcoming.

Discussion has been rewritten and effort has been made to rectify all shortcomings.

10. In the final paragraph of the discussion, consider emphasizing the study's future trends, as well as its strengths and weaknesses.

Limitations, strength and weaknesses of the study are mentioned at end of discussion section.

11. Rewrite the conclusion section in the context of resolving the research problem and clarifying the study's goal achievement.

Conclusion has been rewritten.

12. Updating the reference list and replacing outdated references prior to 2015 with new ones.

New references have been added

Reviewer #2: In the first comment, all pages have no number and no line numbers so it is very difficult to detect the pages in the consort checklist and lines in the manuscript

This manuscript requires a significant amount of improvement, the authors have designed a randomized controlled study to show the effectiveness of the neural mobilization technique on cervical radiculopathy which has already been studied more times in the past by other researchers as the author wrote in the last paragraph of introduction,
¬ I already read reference No 9 in the last paragraph which is completely against the fact you write (Liang, Long PhDa,b; Feng, Minshan PhDa,b; Cui, Xin MSa; Zhou, Shuaiqi MSa,b; Yin, Xunlu PhDa,b; Wang, Xingyu MDc; Yang, Mao MDc; Liu, Cunhuan MDd; Xie, Rong MDa; Zhu, Liguo PhDa,b,∗; Yu, Jie PhDa,b,∗; Wei, ,

Study of Liang et al. concluded that Exercise alone or exercise plus other treatment may be helpful to patients with Cervical radiculopathy. However, exercise option should be carefully considered for each patient with CR. Large-scale studies using proper methodology are recommended that is why in our study carefully designed exercise plan was incorporated in both treatment groups and neural mobilization was additionally added in group 1 to measure its effectiveness. Result of our studies corelate with this study as both groups shows the significant improvement but adding neural mobilization was more helpful in improving pain and functional status.

Introduction

Need editing and rewrite

Introduction section has been edited.

Methods:
¬ What is the novelty of your method and measurement?
¬ The treatment time was very short without follow up

This has been mentioned in the limitations of study.
¬ The arrangement of the method is very weak and you should follow the consort checklist

Statistics
¬ Completely Not clear –please rewrite again and discuss which test you used and why
¬ all tables need to rearrange
¬ Outcomes and estimation need to be explained well

Description of the test used for the analysis is mentioned under the data analysis heading and is as follow:

Normality testing has shown that data were normally distributed for the variable “cervical range of motion” so parametric tests were used for the within (repeated measure ANOVA) and between group comparison

Data were skewed for the variable NPRS and NDI so non parametric tests were used for the within (Friedman Test) and between (Mann Whitney U test) group comparison

Intention to treat analysis with the technique of last observation carried forward (LOCF) was used to handle the missing data due to loss of follow-up.

Discussion:
needs to be as per well defined objectives
Describe sources of potential bias and imprecision
It has to be framed in such a way that readers are able to have good understanding of the current evidences and rationale of the paper

Discussion section has been revised.

6. PLOS authors have the option to publish the peer review history of their article ([what does this mean?](https://journals.plos.org/plosone/s/editorial-and-peer-review-process#loc-peer-review-history)). If published, this will include your full peer review and any attached files.


If you choose “no”, your identity will remain anonymous but your review may still be made public.


**Do you want your identity to be public for this peer review?** For information about this choice, including consent withdrawal, please see our [Privacy Policy](https://www.plos.org/privacy-policy).

Reviewer #1: No

Reviewer #2: No
