# Supplementary material for: Comparison of neural mobilization and conservative treatment on pain, range of motion, and disability in cervical radiculopathy: A randomized controlled trial
Source: PLoS One. 2022 Dec 6;17(12):e0278177. doi: 10.1371/journal.pone.0278177 (PMC9725158; doi:10.1371/journal.pone.0278177)
Supplement: Attachment — Submitted filename: response to reviewer.docx [file pone.0278177.s006.docx]

I am grateful to the reviewers for their insightful comments on the paper. We have been able to incorporate changes to reflect most of the reviewers’ suggestions. The red text is a point-by-point response to the reviewer’s comments and concerns.

 1-Abstract: Provide a brief description of the statistical methods used to compare and express the difference within and between the groups.

A brief description of the statistical method used has been added in the abstract section.

2- Data Analysis section:
a. Mann-Whitney U test is misspelled.

The spelling has been corrected

b. This section is unclear. What comparisons were made with repeated measures ANOVA? With the Friedman tests?

The p-values of the Shapiro-Wilk normality test were greater than 0.05 for cervical ranges but less than 0.05 for NDI and NPRS, showing that data were normality distributed for the cervical range of motions but not normally distributed for NPRS and NDI.

To assess the **difference within the group**, a parametric test i.e., repeated measure ANOVA was used for cervical ranges but its non-parametric counterpart i.e., Friedman test was used for NPRS and NDI.

To assess the **difference between the group**, a parametric test i.e., independent sample t was used for cervical ranges but its non-parametric counterpart i.e., Mann-Whitney U was used for NPRS and NDI.

3- Results section:
a. Specify the type of summary statistic that follows the +/- signs. Both variance and standard deviation are noted in the methods section.

b. Shapiro-Wilks p-values were “greater than” 0.05, would be the standard terminology.

Corrected
c. Be specify, “for mostly” is too vague.

Corrected
d. In the Data Analysis section, state the type of statistical method used to estimate the p-values in Tables 1 and 2. Be transparent.

Table 1 and 2 shows the difference **between** group 1 and 2 at baseline. P values in table 1 are of the Mann Whitney U test and table 2 are of the independent sample t-test.

e. The results show a chi-square result. List and describe the use of this method in the Data Analysis section.

The Chi-square test was not used, table 3 and 4 provide the test statistic (χ^2^) value ("**Chi-square**"), and the significance level ("**Asymp. Sig.**"), which we need to report the result of the **Friedman test.**

f. In the Data Analysis section, list and describe all the statistical methods used.

All the statistical methods used are mentioned in data analysis section

g. Use consistent notation, for instance SD and S.D has been used. The standard is SD.

Corrected

h. All acronyms and abbreviations must be spelled out at first mention.

Corrected
